# Correlation between Pregnancy Related Weight Gain, Postpartum Weight loss and Obesity: a Prospective Study

**DOI:** 10.25122/jml-2019-0015

**Published:** 2019

**Authors:** Roxana Nartea, Brindusa Ilinca Mitoiu, Adriana Sarah Nica

**Affiliations:** 1. National Institute of Rehabilitation and Balneology, Bucharest, Romania; 2. “Carol Davila” University of Medicine and Pharmacy, Bucharest, Romania

**Keywords:** Pregnancy Weight Gain, Postpartum Weight Loss, Body Mass Index, Maternal Obesity, BMI – Body Mass Index, CA – Abdominal Circumcision, IOM – International Institute of Medicine, WHO – World Health Organization

## Abstract

Weight gain during pregnancy can be a real risk factor for long-term obesity which has implications in all areas of medicine. This study is designed to assess pregnancy-related weight gain and postpartum weight loss, to identify a possible correlation between weight gain during pregnancy and the risk of obesity in the late postpartum period. The batch comprised 306 women, hospitalized in the Obstetrics and Gynecology Section of the “Nicolae Malaxa” Clinical Hospital between June - November 2017. During this study, we assessed the weight status using the Weight, Body Mass Index, Height, and Abdominal Circumference. These parameters were clinically assessed in three periods pre-pregnancy, early postpartum period, late postpartum period. We also collected data on the evolution of the pregnancy using the anamnesis and the personal pregnancy monitoring sheet. Pregnancy and postpartum period represent a key moment in women’s lives in which the risk of obesity is real. Understanding women experiences with weight changes during pregnancy and postpartum period can improve the management of losing weight following pregnancy, avoid long-term weight gain and so reduce the risk for obesity. Also, the correct management of obesity should include the assessment of somatic disorders that may cause major dysfunction, requiring complex rehabilitation programs.

## Introduction

Obesity is one of the most challenging global public health issues because it has consequences in all areas of medicine [1]. In Obstetrics, obesity impacts the pregnancy, maternal health but also influences the newborn weight, the weight of the child in infancy and beyond [2]. And last, but not least, somatic consequences that favor the development of chronic algic syndromes with consequences on quality of life. Recent studies show that maternal weight influences the prevalence of obesity in the next generations [1,2]. For many medical health providers, pregnancy and postpartum represent the target period to influence the weight evolution including prevention for future risk of obesity in women [2–4].

During pregnancy and postpartum period, women are frequently in contact with their family doctor and obstetrics specialist medical doctor and due to their concerns about their health and especially for the health of the future baby, they are more open to the promotion of healthy eating and a physical activity routine [1,3,5].

The most basic indicator for obesity is Body Mass Index (BMI, kg/m^2^), which is also accepted as an obesity measure by WHO and the International Institute of Medicine (IOM) [6,7].

From 1990 IOM developed some recommendations for the weight gain during pregnancy in order to optimize fetal growth and postpartum outcome. (Table 1) [8,9].

**Table 1:** Weight Gain during pregnancy according to age

Age	No	Average weight gain during pregnancy	Standard Deviation
<20	23	13,96	5,49
20-25years	81	15,51	9,28
26-30 years	90	14,98	6,52
31-35 years	67	14,13	6,17
>35years	45	12,98	5,99

A medical literature review published in September 2018 shows that in healthy women, diet and physical activity programs in pregnancy and postpartum period can decrease excessive gestational weight gain, the risk of gestational weight gain above IOM recommendation, the prevalence of C section and neonatal respiratory distress syndrome, without any maternal or fetal adverse effects [1,8,9].

**Table:** Table

	BMI	Recommended total gestational weight gain
Underweight	<18,5 kg/m^2^	12,5-18 kg
Normal	18,5-24,5 kg/m^2^	11,5 -18 kg
Overweight	25-29,9 kg/m^2^	7 -11,5 kg
Obesity	≥30 kg/m^2^	5-9 kg

In 2016 a report from Non-Communicable Disease Risk Collaboration was published and it showed that age-standardized prevalence of obesity among women increased from 6.4 in 1975 to 14.9% in 2014 [10–12]. In Europe, according to the same report, maternal obesity ranges from 7% to 25% and is expected to increase to 37% in 2020 [13].

Gestational weight gain is an individualized parameter and its value depends on several factors such as Body Mass before pregnancy, age, parity, ethnicity, smoking, hypertension, and gestational diabetes mellitus [14].

According to more and more recent studies, excessive weight gain during pregnancy is related to overweight during all periods of childhood and also in later life, like 40 years old in daughters [15]. Also, overweight combined with excessive weight gain during pregnancy increases risk of fetal complications and has higher long time likelihood of retaining excessive weight [6,7].

## Aim

The aim of this study is to assess pregnancy-related weight gain and postpartum weight loss to identify a possible correlation between weight gain during pregnancy and the risk of obesity in the late postpartum period.

## Material and Method

This observational prospective study took place at “Nicolae Malaxa” Clinical Hospital during June- November 2017 stage one, respectively September 2017- January 2018 for the second stage.

The patients included in the study were selected in accordance with the Ethical Principles from the Helsinki Declaration of Human Rights, in accordance with the Good Practice Rules in the Clinical Study and the current legal recommendations.

Inclusion criteria: patient admitted in Obstetrics Department of Nicolae Malaxa Hospital; a desire to participate in the study; informant Agreement Form signed; knowledge of the Romanian Language at a native level; term delivery; vaginal or C-section delivery without complications

Exclusion criteria: patient refusal to participate in the study; vaginal or C-section birth with complications; delivery before term.

This study includes 306 women, hospitalized in the Obstetrics and Gynecology Section of the “Nicolae Malaxa” Clinical Hospital between June - November 2017. During the study, we assessed the weight status using the Weight (kg), Body Mass Index (BMI= Weight (kg)/ height (m)2), Height (m) and Abdominal Circumference (measured with a tailoring meter on umbilical level). These parameters were clinically assessed in three moments pre-pregnancy, early postpartum period (3-5 days after delivery), late postpartum period (6 weeks after delivery). We also collected data on the evolution of the pregnancy using the anamnesis and the personal pregnancy monitoring sheet (patient age, education level, age of the pregnancy, pregnancy complications, and maternal medical history). If in the early postpartum stage we have 306 patients, in the late postpartum period only 236 women evaluated, 57 did not show up for the second part of the study. For statistical analysis we used Microsoft Excel and SPSS 2000- IBM program. In data processing we take in count the following rules: quantitative variables were assessed according to distribution, dispersion indicators (minimum and maximum value, standard deviation) and central trend indicators (average and median) and in case of qualitative variables we calculated absolute and relative frequencies. We have made comparisons according to the type of birth. For the continuous quantitative variables, we used the T-Student Test.

## Results

The results obtained in our group showed, on one hand, the high percentage of caesarean birth, namely 62.75%, but also a large number of adolescent mothers, respectively 5.7%.

The 306 women included in the study had at the time of inclusion in the study ages between 14 and 43 years, with an average age of 28.5 ± 6.37. More than half of the patients included in the study were primiparous (52.94%) and 39.22% were at the second pregnancy.

Weight gain during pregnancy ranges between -15kg and +40Kg, averaging 14.3±6.28 kg. According to Body Mass Index, if in the pre-pregnancy period the percentage of overweight women was 22.22% and the obese 6.86%, in the late postpartum period these percentage increases to 29.66% for overweight mothers and to 13.98% for the obese ones.

Average pre-pregnancy Body Mass Index is 23.03 ± 4.01 kg/m^2^ with a minimum of 15.31 kg/m^2^ and a maximum of 38.86 kg/m^2^.

Weight gain during pregnancy in our study is not statistically significant according to age. Adolescent mums had an average weight gain 13.98±5.49 kg and women older than 35 years old had an average weight gain of 12.98±5.99 kg. (Table 1).

**Figure 1:**
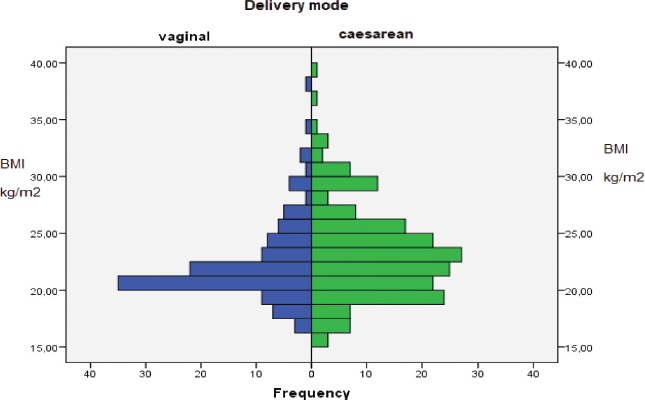
BMI and delivery mode

Pre-natal BMI in women who delivered via ceasarean (23.38 ± 4.18 kg/m^2^) is statistically significantly higher (p= 0,048) compared to those with vaginal birth (22.44±3.64 kg/m²). (Figure 1)

The abdominal circumference in early postpartum varies between 69 cm and 136 cm; the average is 101.59 ±10.15 cm. In early post-partum abdominal circumference is statistically significantly higher (p= 0.002) in case of women who delivered via caesarean section (102.96 ± 10.35 cm) than those with vaginal birth (99.27 ± 9.38 cm).

**Figure 2:**
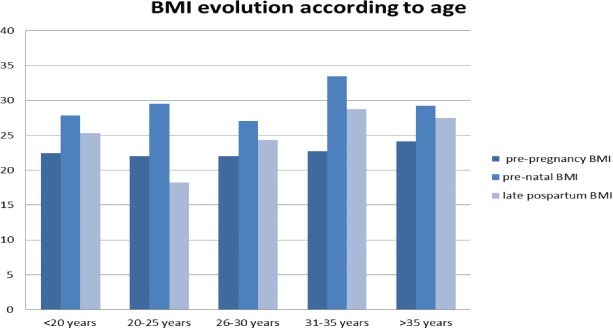
IMC evolutions according to age

In late postpartum, a weighing difference of -50 kg to +5 kg was recorded in relation with early postpartum weight, with an average of -9.31± 5.16 kg.

In relation to pre-pregnancy BMI, in the late postpartum period in our group was recorded a weight difference between -11 kg and +30 kg, average being 5.15 ± 5.47 kg.

Weight gain during pregnancy, weight in late postpartum compared with pre-pregnancy weight did not show statistically significant differences due to delivery mode.

The abdominal circumference in late-postpartum is statistically significantly higher (p=0.009) in case of women with C section (89.77 ± 10.33 cm) than those with vaginal birth (86.17 ± 10.09 cm).

**Table 2:** Weight status – parameters

	Delivery mode	N0	Average	Standard deviation	P value
IMC (kg/m^2^)	Vaginal birth	114	22.44	3.64	0.048
	Caesarean	192	23.38	4.18	
Weight grow during pregnancy (kg)	Vaginal birth	114	14.32	5.18	0.973
	Caesarean	192	14.29	5.86	
Pre-natal abdominal circumference	Vaginal birth	114	99.27	9.38	0.002
	Caesarean	191	102.96	10.35	
Weight loss in late postpartum	Vaginal birth	91	–9.16	4.29	0.716
	Caesarean	145	–9.41	5.64	
Weight loss in late post versus pre-pregnancy	Vaginal birth	91	5.59	5.18	0.323
	Caesarean	145	4.87	5.64	
Late postpartum abdominal circumference	Vaginal birth	91	86.17	10.09	0.009
	Caesarean	145	89.77	10.33	

The weight increase in pregnancy correlates negatively statistically significant (rho = -0.142; p = 0.013) with the pre-pregnancy body mass index, the smaller the pre-pregnancy index is, the greater the weight increase in pregnancy, and vice versa.

Underweight patients (BMI<18,5 before pregnancy) had a medium weight gain of 14.67 ± 5.3 Kg during pregnancy. From them, in late postpartum, the average medium weight gain was 6.19 ± 5.16 kg, which means that more than 50% (15 out of 21) of them are recorded now as normal weight.

Women with normal BMI in pre-pregnancy had during pregnancy a medium weight gain of 14.56 ± 5.74 kg and the residual weight gain in late postpartum of 5.55 ± 5.61 kg. In this category, 46% of them in late postpartum are overweight.

The overweight women had a medium weight gain during pregnancy of 14.25 ± 7,3 kg, higher than the recommendations of IOM. In late postpartum residual weight gain was 3.46 ± 4.6Kg and 34.14% of them had in late postpartum an IMC higher than 30.

The women with an IMC higher than 30 before pregnancy, had a medium weight gain during pregnancy of 11.17 ± 4.89 kg (also greater than the IOM recommendations) and a residual weight gain in late postpartum of 2.06 ±7.75Kg.

The weight increase in pregnancy correlates negatively statistically significant (rho = -0.118; p = 0.039) with the number of pregnancies, so if the weight increase in pregnancy is less the higher the number of pregnancies are, and vice versa.

The weight increase in pregnancy correlates positively statistically significant with the duration of gestation (rho = 0.140; p = 0.014) and the weight of the newborn (rho = 0.163; p = 0.004), so if the weight increase in pregnancy is higher gestation duration is; respectively the weight of the newborn was higher and vice versa. (Table 3)

**Table 3:** Correlation of ponderal status indicators

		CA early postpartum	Weight loss in postpartum	Weight gain (G late postpartum – G before pregnancy	CA late postpartum	Age	Number of pregnancies	Education level
Weight loss in postpartum	rho	-.140*		.435**	–.103	.035	–.015	.101
	P	.032		.000	.159	.594	.814	.122
	N	236		236	188	236	236	236
Weight gain (G late postpartum – G before pregnancy	rho	.153*	.435**		.126	.042	–.122	.057
	P	.019	.000		.086	.521	.061	.385
	N	236	236		188	236	236	236
CA late postpartum	rho	.831**	–.103	.126		.370**	.162*	.145*
	P	.000	.159	.086		.000	.012	.025
	N	237	188	188		237	237	237
Age	rho	.241**	.035	.042	.370**		.415**	.423**
	P	.000	.594	.521	.000		.000	.000
	N	305	236	236	237		306	305
Number of pregnancies	rho	.139*	–.015	–.122	.162*	.415**		-.135*
	P	.015	.814	.061	.012	.000		.019
	N	305	236	236	237	306		305
Education level	rho	.073	.101	.057	.145*	.423**	–.135*	
	P	.202	.122	.385	.025	.000	.019	
	N	305	236	236	237	305	305	

Early postnatal abdominal circumference correlates positively statistically significant with the weight difference in late postpartum versus pre-pregnancy weight (rho = 0.153; p = 0.019) with abdominal circumference at 6-8 week postnatal (rho = 0.831; p <0.001 ), with the number of pregnancies (rho = 0.139, p = 0.015), gestation duration (rho = 0.183, p = 0.001) and the weight of the newborn (rho = 0.210 p <0.001), that means, the higher the prenatal abdominal circumference, the difference in weight at 6-8 weeks relative to baseline weight, abdominal circumference at 6-8 weeks post-natal, age, number of pregnancies, duration of gestation and the weight of the newborn were higher, and vice versa.

Prenatal abdominal circumference correlates negatively statistically significant with weight difference at 6-8 weeks relative to pregnancy weight (rho = -0.140; p = 0.032), so the higher the prenatal abdominal circumference, the difference in weight at 6-8 weeks compared to pregnancy weight was higher, and vice versa.

The difference in weight at 6-8 weeks of pregnancy weight correlates positively statistically significant with the difference in weight at late postpartum relative to baseline weight (rho = 0.435; p <0.001), so, if the difference in weight at 6-8 weeks versus the weight in pregnancy was higher, the difference in weight during late postpartum relative to the initial weight was higher, and vice versa. Abdominal circumference at 6-8 weeks post-partum correlates positively statistically significant with age (rho = 0.370; p <0.001), with the number of pregnancies (rho = 0.162; p = 0.012), with the education level (rho = 0.145; p = 0.025) and the weight of the newborn (rho = 0.139; p = 0.033), i.e. the abdominal circumference at 6-8 weeks postnatal was higher in both the age, the number of pregnancies, the level of education and the weight of the newborn were larger, and vice versa.

## Conclusion

Pregnancy and postpartum period represent a key moment in women’s lives in which the risk of obesity is real.

Understanding women experiences with weight changes during pregnancy and postpartum period can improve the management of losing weight following pregnancy, avoid long term weight gain and so reduce the risk of obesity. Also, the correct management of obesity should include the assessment of somatic disorders that may cause major dysfunction, requiring complex rehabilitation programs.

## Conflict of Interest

The authors confirm that there are no conflicts of interest.
